# Novel Anti-Inflammatory Therapies in Coronary Artery Disease and Acute Coronary Syndromes

**DOI:** 10.3390/life13081669

**Published:** 2023-07-31

**Authors:** Yannis Dimitroglou, Constantina Aggeli, Panagiotis Theofilis, Panagiotis Tsioufis, Evangelos Oikonomou, Christos Chasikidis, Konstantinos Tsioufis, Dimitris Tousoulis

**Affiliations:** 1 First Department of Cardiology, “Hippokration” General Hospital, University of Athens Medical School, 11527 Athens, Greece; dimiyann@hotmail.com (Y.D.); dina.aggeli@gmail.com (C.A.); ptsioufis@gmail.com (P.T.); ktsioufis@gmail.com (K.T.); drtousoulis@hotmail.com (D.T.); 2 Third Department of Cardiology, Thoracic Diseases General Hospital “Sotiria”, University of Athens Medical School, 11527 Athens, Greece; boikono@gmail.com; 3 Department of Cardiology, General Hospital of Corinth, 20100 Corinth, Greece; chasikid@otenet.gr

**Keywords:** inflammation, atherosclerosis, cytokines, vascular inflammation, acute coronary syndromes, endothelial dysfunction, interleukin, treatment

## Abstract

Evidence suggests that inflammation plays an important role in atherosclerosis and the consequent clinical presentation, including stable coronary artery disease (CAD) and acute coronary syndromes (ACS). The most essential elements are cytokines, proteins with hormone-like properties that are produced by the immune cells, endothelial cells, platelets, fibroblasts, and some stromal cells. Interleukins (IL-1β and IL-6), chemokines, interferon-γ (IFN-γ), and tumor necrosis factor-alpha (TNF-α) are the cytokines commonly associated with endothelial dysfunction, vascular inflammation, and atherosclerosis. These molecules can be targeted by commonly used therapeutic substances or selective molecules that exert targeted anti-inflammatory actions. The most significant anti-inflammatory therapies are aspirin, statins, colchicine, IL-1β inhibitors, and IL-6 inhibitors, along with novel therapies such as TNF-α inhibitors and IL-1 receptor antagonists. Aspirin and statins are well-established therapies for atherosclerosis and CAD and their pleiotropic and anti-inflammatory actions contribute to their efficacy and favorable profile. Colchicine may also be considered in high-risk patients if recurrent ACS episodes occur when on optimal medical therapy according to the most recent guidelines. Recent randomized studies have also shown that therapies specifically targeting inflammatory interleukins and inflammation can reduce the risk for cardiovascular events, but these therapies are yet to be fully implemented in clinical practice. Preclinical research is also intense, targeting various inflammatory mediators that are believed to be implicated in CAD, namely repeated transfers of the soluble mutant of IFN-γ receptors, NLRP3 inflammasome inhibitors, IL-10 delivery by nanocarriers, chemokine modulatory treatments, and reacting oxygen species (ROS) targeting nanoparticles. Such approaches, although intriguing and promising, ought to be tested in clinical settings before safe conclusions can be drawn. Although the link between inflammation and atherosclerosis is significant, further studies are needed in order to elucidate this association and improve outcomes in patients with CAD.

## 1. Introduction

Coronary artery disease (CAD) is a leading cause of morbidity and mortality worldwide, affecting millions of people each year [1]. Despite advances in pharmacological and interventional management, CAD remains a major challenge for healthcare providers, highlighting the need for new and effective therapies to improve outcomes, such as anti-inflammatory agents.

The link between inflammation and atherosclerosis was first proposed in the 19th century by German pathologist Rudolf Virchow who observed the presence of immune cells in the arterial walls of patients with atherosclerosis [2]. In the 1980s and 1990s, studies showed that markers of inflammation, such as C-reactive protein (CRP), were elevated in patients with CAD and could be used to predict the risk of future cardiovascular events [3]. More recent research also showed that cytokines interact to initiate and sustain inflammation in the atherosclerotic plaque and could contribute to the inflammatory process [4]. This research has led to the development of new therapies targeting inflammation in CAD, such as anti-inflammatory drugs and immunomodulating therapies [5].

This review aims to describe the pathophysiological pathways between CAD and inflammation, present established and novel anti-inflammatory agents used therapeutically in patients with CAD, and identify areas for future research.

## 2. Inflammatory Mechanisms in Atherosclerosis and Coronary Artery Disease

Atherosclerosis is a multifactorial process and can be triggered by genetic susceptibility as well as cardiovascular risk factors like hypertension, diabetes mellitus, and smoking [6]. Underlying mechanisms include, among others, endothelial dysfunction and abnormal lipid metabolism, which are induced by inflammation [7].

Increased oxidative stress can promote endothelial dysfunction, which is the initial step of atherogenesis [8]. Nitric oxide (NO) plays a critical role in the regulation of endothelial function as it possesses vasorelaxant, anti-thrombotic, anti-proliferative, and anti-inflammatory properties [9]. The underlying mechanisms of endothelial dysfunction include reduced bioavailability of NO due to increased degradation and diminished production. When endothelial cells become activated, they can promote vasoconstriction, thrombosis, leukocyte mobilization and migration, and vascular smooth muscle cell (VSMC) proliferation [5,8]. The result of endothelial dysfunction is the production of reactive oxygen species (ROS), which induce macrophage accumulation and cell apoptosis, causing even further endothelial dysfunction [10].

The influx of low-density lipoprotein (LDL) particles, especially small-dense LDL, is no longer considered a passive process and is facilitated by transcytosis mechanisms [11]. After retention in the vessel wall, LDL undergoes various modifications and can bind to lectin-like oxidized LDL receptor-1 (LOX-1) located on the surface of VSMC and macrophages, leading to the formation of foam cells [12]. Moreover, LDL particles can form complexes with proteoglycans and glycosaminoglycans, promoting immune responses and cholesterol crystal formation [13]. Other lipoproteins such as lipoprotein-a (Lp-a), lipoprotein-associated phospholipase A2, and high-density lipoprotein (HDL) have also been associated with pro-inflammatory and anti-inflammatory properties, respectively [14,15,16]. In particular, the anti-inflammatory role of HDL cholesterol has been recognized lately, and indices such as monocyte-to-HDL-C ratio are explored to improve the risk estimation of CAD prognosis [17,18].

The immune system, including both innate and adaptive immunity, can enhance atherogenesis, and lately, there is an increasing interest in the role of different cell types of the immune system [19]. There is also an increased interest in cardiac-specific or inflammatory molecules that can be used either as reliable biomarkers for early detection and counteraction of the inflammatory process or as specific therapeutic targets [20]. Cytokines are proteins with hormone-like properties that are soluble and mediate the communication among immune cells or between immune cells and target organs [5]. They are produced by immune cells, endothelial cells, platelets, fibroblasts, and some stromal cells. Interleukins (ILs), chemokines, interferon-γ (IFN-γ), and tumor necrosis factor (TNF)-α, are the cytokines commonly associated with atherosclerosis [5,21].

IL-1 has two subtypes, IL-1α and IL-1β. IL-1β is produced mainly by macrophages, endothelial cells, and adipocytes and exerts its actions by binding to specific receptors, including IL-1 receptor, toll-like receptor (TLR) 2, and TLR4, and activating downstream signaling pathways, such as the nuclear factor kappa-light-chain-enhancer of activated B cells (NF-κΒ) and mitogen-activated protein kinases (MAPK) [22,23,24,25]. NF-κΒ then promotes the formation of other cytokines, such as IL-6 and TNF-α, and the transmigration of leucocytes mediated by a variety of adhesion molecules, such as intercellular adhesion molecule-1 (ICAM-1), vascular cell adhesion protein-1 (VCAM-1), P-selectin, and E-selectin, or chemokines, such as chemokine (C-C motif) ligand CCL2, also known as Monocyte Chemoattractant Protein-1 (MCP-1) [25,26]. IL-1β is produced after the precursor molecule is cleaved by the enzyme caspase-1. Caspase-1, in turn, is activated by the NLRP3 inflammasome [27]. Finally, various pathogen-associated molecular patterns (PAMPs) and damage-associated molecular patterns (DAMPs) as well as reduced energy levels and mitochondrial and lysosomal dysfunction can activate the NLRP3 inflammasome [28]. The inflammasome is also responsible for the increased activation of IL-18, which can promote endothelial dysfunction and increase downward cytokine production [29]. Cohort and prospective studies have shown that the concentration of NLRP3 inflammasome, IL-1β, and IL-18 is increased in the atheromas and monocytes of the peripheral blood of patients with CAD and especially those with acute coronary syndromes (ACS) when compared to the control groups [30,31]. Of interest, NLRP3 inflammasome and, as a result, IL-1β, are also partly modulating the secretion of proprotein convertase subtilisin/kexin type 9 (PCSK9), a well-established therapeutic target in CAD [32].

Interleukin-6 (IL-6), produced after the activation of IL-1β or ΤΝF-α, is secreted by macrophages, monocytes, fibroblasts, and endothelial cells, and has pro-inflammatory and pro-thrombotic properties [33,34]. Preclinical studies in murine models have shown that IL-6 promotes atherosclerosis and possibly plaque vulnerability and that injection of IL-6 results in increased CRP, TNF-α, and fibrinogen levels [35,36,37]. In addition, the findings of a large Mendelian randomization study indicated a potentially causative role in the pathophysiology of atherosclerosis [38]. However, a lifetime IL-6 deficiency, as tested in double knockout (ApoE^−/−^-IL-6^−/−^) mice, could possibly enhance the development of atherosclerosis. These observations suggest that low but existent baseline levels of IL-6 are required to modulate vascular inflammation [39].

Interleukin-10 (IL-10) is an anti-inflammatory cytokine produced by monocytes and Treg cells [40,41]. It exerts its role by modulating inflammatory properties of IL-1, IL-6, and TNF-α and by suppressing monocytes, macrophages, and T-cells. Moreover, IL-10 promotes lipid uptake by macrophages and has a beneficial role in the metabolism of cholesterol. Regarding its role in atherosclerosis, data from in vitro and in vivo studies support that IL-10 prevents the progression of atherosclerotic plaque and reduces plaque instability and injury caused by reperfusion [42,43]. However, a study that included 930 patients from the Multiethnic Study of Atherosclerosis (MESA) found that IL-10 levels were not related to cardiovascular events. In addition, IL-10 was a poor biomarker regarding subclinical atherosclerosis [44].

Chemokines are secreted cytokines with the ability to induce chemotaxis and contribute to the initiation and maintenance of the inflammatory process. CCL-2 (or MCP-1) is a chemokine known to attract inflammatory cells, such as monocytes and macrophages in the intima, leading to the production of IL-6 [45]. A study using CCL2-deficient LDLr^−/−^ mice on a high-cholesterol diet revealed significantly reduced lipid deposition in the aortas of CCL2-deficient mice along with lesser macrophage infiltration into the aortic walls compared to the control mice [46]. Another study confirmed these findings and showed that CCR2-deficient ApoE^−/−^ mice exhibited significantly reduced lipid deposition without affecting the lipid profile [47]. A more recent study found that a dietary intervention based on the DASH diet was associated with reduced CCL-2 concentration which in turn was associated with a decrease in the volume of atheromas as determined by computed tomography [48]. CCL-5, also known as RANTES, is a platelet-derived chemokine that promotes monocyte recruitment at the site of vascular injury [49,50]. A study performed on 1769 subjects from the Atherosclerosis Risk in Community (ARIC) study found that RANTES was positively associated with total wall volume, maximum wall thickness, mean minimum fibrous cap thickness, and hs-CRP, indicating associations between RANTES and the extent of atherosclerosis [51].

IFN-γ is a modulatory cytokine with both pro and anti-inflammatory potential [52]. IFN-γ participates in the initiation of atherosclerosis since it promotes endothelial dysfunction, VSMC proliferation, leukocyte mobilization, and foam cell accumulation [53]. IFN-γ can upregulate cytokines such as IL-1β, IL-6, and CCL5, shifting macrophages towards the atheroprone M1 phenotype and T cells towards a Th1 phenotype. A study performed in ApoE^−/−^ mice found that the additional deficiency in IFN-γ led to a reduction in atherosclerotic lesion size, lipid accumulation, and lesion cellularity with a marked increase in lesion collagen content [54]. Similar data have also been reported in other preclinical studies [55,56]. In addition, IFN-γ levels, as well as other indices such as the IFN-γ-to-IL-10 ratio, have been associated with the extent of calcification in patients with chronic CAD [57]. However, there is a relative scarcity of epidemiological studies with sufficient evidence to verify whether these mechanisms remain significant in human subjects.

TNF-α can be produced by most cell types and acts by inducing the expression of other proatherogenic cytokines [58]. The atherogenic role of TNF-α was highlighted in an experimental study in which mice deficient in both apoE and TNF-α (apoE^−/−^/TNF-alpha^−/−^) were compared with apoE^−/−^ mice. Serum total cholesterol levels did not differ in the two groups, but the atherosclerotic plaque area in the aorta was smaller in the first group. Further analysis indicated that the expression levels of ICAM-1, VCAM-1, and MCP-1, together with the oxidized LDL uptake levels were also significantly lower [59]. In addition, according to a meta-analysis of the available genetic studies, specific gene polymorphisms in the TNF-α locus have been associated with susceptibility to CAD [60]. Interestingly, TNF-α was a significant predictor of cardiovascular events according to an algorithm developed by machine learning using data from the MESA [61].

It is also worth noting, from a pathophysiological point of view, that atherosclerosis is not synonymous with CAD, especially with the increased incidence of ACS. There are also pathologic differences between atherosclerosis of the coronary vessels and the peripheral arteries leading to different mechanisms of acute events [62]. Imaging studies have provided evidence that obstructive lesions of the coronary vessels may remain clinically silent, while non-obstructive lesions may provoke ACS. The local characteristics leading to plaque destabilization and eruption vary from anatomic and hemodynamic characteristics to ones closely related to vascular inflammation [63]. There is, therefore, a pathophysiological basis that anti-inflammatory therapies may not only halt the development and progression of plaques but also decrease the likelihood of provoking ACS.

## 3. Anti-Inflammatory Therapies in CAD

**Figure 1:**
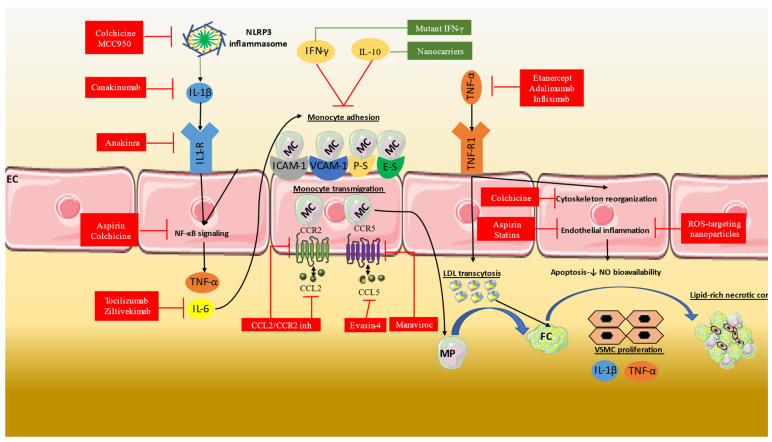
Inflammatory mechanisms involved in atherosclerosis and the mechanisms of action of significant anti-inflammatory therapies. The most significant mechanisms include endothelial dysfunction, increased transmigration of monocytes, LDL transcytosis with VSMC proliferation, and lipid-rich necrotic core formation as well as the inflammasome and NF-κB mediated IL-1, IL-6, and TNF-α activation. The available anti-inflammatory therapies are either non-selective, such as aspirin and colchicine, or those that selectively antagonize these mechanisms as is the case of IL-1b and IL-6 inhibitors. CCL2: chemokine (C-C motif) ligand; CCR: CCR: C-C chemokine receptor; IL: interleukin; IFN: interferon; ICAM: intercellular adhesion molecule; FC: foam cell; MC: monocyte; MP: macrophage; NO: nitric oxide; ROS: reactive oxygen species TNF: tumor necrosis factor; VCAM: vascular cell adhesion protein; VSMC: vascular smooth muscle cell.

The main pathophysiological mechanisms of action of the most common anti-inflammatory therapies are presented in Table 1. The use of anti-inflammatory therapies in clinical practice includes the pleiotropic actions of aspirin, statins, and colchicine (Figure 1). Large randomized clinical studies and meta-analyses have shown that these therapies have favorable anti-inflammatory effects and can reduce the hazard of cardiovascular events (Table 2).

### 3.1. Aspirin

At higher doses, aspirin functions as an anti-inflammatory drug by inhibiting cyclooxygenase (COX) and pro-inflammatory signaling pathways such as NF-κB. At a lower dose, aspirin provides cardioprotective benefits by inhibiting thromboxane B2, which is an eicosanoid that promotes platelet activation [76]. However, there are sufficient data indicating the anti-inflammatory role of aspirin, even at lower doses [77].

The activation of the Thromboxane prostanoid (TP) receptor is involved in the pathogenesis of oxidative stress and vascular inflammation [78]. The underlying pathophysiological mechanisms include the expression of pro-inflammatory markers by endothelial cells, VSMC proliferation, and the production of prostanoids and isoprostanes [79,80,81]. In addition, increased concentration of thromboxane synthase has been associated with increased presence of inflammatory cells in the atherosclerotic plaque [82]. It has been shown that EV-077, a TP receptor antagonist and thromboxane synthase inhibitor, can reduce endothelial inflammation and VSMC proliferation [83]. It is not yet clear if low-dose aspirin can sufficiently inhibit such mechanisms [84,85].

The effect of aspirin on the reduction of CAD risk is believed to be higher in patients with increased markers of inflammation. This was evident according to an analysis of more than 1000 subjects participating in the Physicians’ Health Study. In this randomized control trial for aspirin versus placebo, the effect of aspirin in risk reduction was more robust in patients with higher baseline levels of CRP [64]. Aspirin has also been shown to be associated with a reduction in CRP levels. A study measured CRP at baseline and three months after an ACS event and found that lower aspirin dose and poor adherence to therapy were associated were relatively more increased levels of CRP at the follow-up. However, there were not sufficient data to determine whether this association is causative [65].

Other inflammatory markers, such as IL-6 and TNF-α, which are elevated in the plasma or at the ruptured atherosclerotic plaques, could be lowered by aspirin. In one such study, IL-6 and TNF-α were decreased two weeks after aspirin initiation at 300 mg. At lower doses, the decrease was not statistically significant [86]. In patients with stable CAD, the levels of TNF-α were decreased one year after initiation of aspirin, according to a study that randomized patients to aspirin or clopidogrel. However, TNF-α levels were also decreased in the clopidogrel group, and there was no significant difference in the two arms [86]. Similar results were also reported in a randomized trial conducted in patients with myocardial infarction [87].

A placebo-controlled clinical study compared macrophage-colony-stimulating factor (M-CSF), CRP, and urinary 11-dehydro-thromboxane B2 levels in patients with stable CAD after initiation of 300 mg aspirin. They reported a significant reduction of the aforementioned molecules. However, because M-CSF remained related to 11-dehydro-thromboxane B2, they concluded that the inhibition was not adequately strong [88].

Aspirin can also exhibit an anti-inflammatory effect through mechanisms involving vascular inflammation. Aspirin can increase the activity of platelet and endothelial NO synthase [89,90]. The effect on platelet NO synthase is COX-independent in the acute phase but COX-dependent in the chronic phase [90]. A prospective study examined the anti-inflammatory effects of low-dose aspirin in healthy male volunteers with induced skin blisters. The results showed that aspirin reduced leukocyte accumulation through the synthesis of and the up-regulation of the ALX receptor. This effect was attributed to the activation of antiadhesive NO, which inhibited interactions of leukocytes and endothelial cells and subsequent extravascular leukocyte migration [91]. Similar results were reported in a study that measured 15-epi-lipoxin A4 levels after initiation of aspirin in healthy volunteers and in doses down to 81 mg [92]. Another study, in a mouse model of mesenteric ischemia, showed that aspirin reduced neutrophil adhesion and transmigration by increasing the formation of 15-epi-LXA4. Aspirin triggers 15-epi-LXA4 in healthy humans at an oral dose of 81 mg/day [93]. Apart from the aforementioned mechanisms, a cross-sectional study investigated the levels of myeloid-related protein (MRP)-8/14, a molecule that regulates vascular inflammation in patients with ACS, and found that patients on aspirin had significantly lower MRP8/14 levels [94]. Such findings could potentially explain the results of a randomized placebo-controlled trial involving volunteers, which reported improved endothelial function of the patients receiving aspirin compared to those on placebo [95].

### 3.2. Statins

Statins are used primarily for reducing LDL by the inhibition of the enzyme 3-hydroxy-3-methylglutaryl coenzyme A (HMG-CoA) reductase, which is responsible for the transformation of HMG-CoA to mevalonate [96]. Primarily because of the reduction of LDL cholesterol, they have been shown to reduce mortality, especially in high-risk patients [97]. However, their pleiotropic actions, including their anti-inflammatory potential, are both significant and well-recognized [98,99,100]. Statins have a beneficial role in plaque stabilization by improving endothelial function and by decreasing oxidative stress and inflammation [101]. Several pathophysiological mechanisms have been proposed as potential mediators of these effects since the mevalonate pathway also influences endothelial function, inflammatory response, and coagulation [102]. A meta-analysis of 15 randomized controlled trials showed that statins reduce the levels of circulating endothelin-1 (ET-1), an effect not affected by the dose or the duration of the statin use. Statin lipophilicity, however, was shown to modify the ET-1 reduction [67]. Statins also affect endothelial and microvascular function because they reduce circulating levels of asymmetrical dimethyl arginine (ADMA), an inhibitor of the NO synthase [103]. Animal models have shown that atorvastatin can reduce the levels of TNF-α, IL-6, and MCP-1 and increase the levels of IL-10 [104]. Another animal study showed that a hypercholesterolemic diet increases the expression of specific TLR mRNA, an effect that can be inhibited by fluvastatin [105]. The thickening of the fibrous cap and the macrocalcification are also involved in the stabilization of the atherosclerotic plaques [106]. Specifically, according to the findings of a study that used optical coherence tomography (OCT), the increase in the fibrous cap was associated with decreased inflammatory biomarkers. The effects of atorvastatin were dose-related, being higher at 20 mg/day when compared to 5 mg/day [107].

JUPITER was a randomized-control trial that recruited more than 17,000 apparently healthy subjects with LDL more than 130 mg/dl and high sensitivity CRP of more than 2 mg/L who were randomized to either rosuvastatin 20 mg daily or placebo. Rosuvastatin reduced both the LDL and the CRP levels by 50% and 37%, respectively, and was associated with reduced hazard for myocardial infarction, stroke, cardiovascular death, and all-cause mortality [66]. In addition, two meta-analyses performed in human subjects confirmed that statin and ezetimibe combination therapy can reduce hs-CRP, IL-6, and IFN-γ levels. Results regarding TNF-α and MCP-1 were non-significant [68,69]. Interestingly chronic kidney disease may potentiate the anti-inflammatory role of statins. A randomized, double-blind, cross-over placebo-controlled trial found that simvastatin, with or without the addition of ezetimibe, can reduce the levels of IFN-γ and MCP-1 in patients with diabetes and chronic kidney disease, but not in patients who had only diabetes [108].

### 3.3. Colchicine

Colchicine is a well-known drug for its anti-inflammatory properties and has been used to treat acute gout for over a thousand years. There is also increasing interest in its utility for the prevention and treatment of various cardiovascular diseases [109]. Colchicine exerts its anti-inflammatory properties primarily by binding to tubulin and inhibiting its polymerization. This property disrupts the function of the cytoskeleton, reducing chemotaxis, migration, and cell signaling and inhibits cell mitosis [110]. Other mechanisms involved are the decreased expression of adhesion molecules, downregulation of the TNF-α modulated NF-κB pathway, and the suppression of inflammasome formation [110,111]. The latter has been shown to be a significant pathophysiological mechanism in patients with both acute and chronic coronary syndromes [112,113].

Several clinical studies have investigated the role of colchicine in cardiac remodeling immediately following a myocardial infarction (MI). An initial randomized study performed in patients with ST-elevation MI showed that colchicine administration for five days immediately after primary percutaneous coronary revascularization resulted in reduced creatine kinase-myocardial-brain fraction concentration and reduced infarct size as assessed by magnetic resonance imaging (MRI) [114]. Contrary to the aforementioned study, a more recently published randomized controlled trial that tested the effects of high-dose colchicine at the time of revascularization after ST-elevation MI on infarct size as assessed by MRI did not report significantly improved LV remodeling or reduced infarct size [115]. Interestingly, even in patients referred for coronary revascularization, preprocedural administration of colchicine, reduced inflammation markers such as CRP and IL-6, but did not affect the risk for percutaneous coronary intervention (PCI)-related MI [116]. In another relevant randomized study, colchicine was administrated 18 h before the planned revascularization. The levels of pre-PCI cytokines were lower in the treatment group, but the respective levels did not differ at 38 h after colchicine administration [117].

A randomized placebo control trial (COLCOT trial) assigned 4745 patients within 30 days after an MI to either colchicine 0.5 mg daily or placebo. The primary endpoint of cardiovascular death, resuscitated cardiac arrest, myocardial infarction, stroke, or urgent hospitalization for angina leading to coronary revascularization was met significantly less often in the colchicine than in the placebo group (HR: 0.77, 85% CI 0.61–0.91, *p* = 0.02). Regarding adverse effects, pneumonia was reported significantly more often in patients assigned to the colchicine group [70]. Smaller randomized studies have also shown similar favorable results [118,119].

Another large-scale randomized placebo-controlled trial (LoDoCo-2) assigned 5522 patients with chronic CAD to either colchicine 0.5 mg/day or placebo. The primary endpoint was the composite of cardiovascular death, nonprocedural MI, ischemia-driven coronary revascularization, or ischemic stroke. According to the results of the study, the primary endpoint occurred in significantly fewer patients in the colchicine group than in the placebo group (HR: 0.72, 95% CI 0.57–0.92, *p* = 0.007). However, the incidence of death from non-cardiovascular causes was higher in the colchicine group [71]. The favorable effect of colchicine as a preventive medication in chronic coronary syndromes has also been shown in smaller randomized trials and in relevant meta-analyses [120,121,122].

### 3.4. IL-1b Inhibition

The anti-inflammatory role of IL-1β in CAD has been known for some years, but the potential for selective inhibition clinically applicable to CAD was not achieved until recently. Canakinumab is a human monoclonal antibody first approved for the treatment of various autoimmune and autoinflammatory disorders [123]. Canakinumab reduces plasma levels of IL-6 and hs-CRP with only modest effects on the lipid profile [124]. Thus, it was hypothesized that its potential effect on cardiovascular events would be independent of lipid-level lowering.

The CANTOS study was a randomized, double-blind, placebo-controlled clinical trial that investigated the effect of canakinumab in reducing the recurrence of cardiovascular events. A total of 10,061 patients with a medical history of MI and hs-CRP ≥ 2 mg/L were assigned to either canakinumab at a dose of 50, 150, or 300 mg every three months subcutaneously or placebo. The primary endpoint was the composite of cardiovascular death, nonfatal myocardial infarction, or nonfatal ischemic stroke. Canakinumab reduced the initially increased hs-CRP levels, with reductions being dose-dependent. Among the different doses, only 150 mg reached the prespecified level of statistical significance for both the primary endpoint and the secondary endpoint of hospitalization for unstable angina that led to coronary revascularization (HR: 0.83, 95% CI 0.73–0.95, *p* = 0.005) [73]. Further analysis of the available data, which calculated the total number of events, showed that the decrease of the cardiovascular hazard was significant for all doses of canakinumab when compared to placebo. Interestingly, the reduced hazard was observed independently of the hs-CRP or IL-6 levels [125]. However, in the CANTOS trial, canakinumab was associated with an increased risk for fatal infections, and there was a non-significant difference regarding all-cause mortality [73]. The only modest decrease in the hazard for cardiovascular death, in conjunction with the increased risk for complications and the non-significant effect on all-cause mortality, raised the need for the development of other novel therapeutic strategies as well as for an improved selection of the patients who are more likely to respond to such therapies [126].

### 3.5. IL-6 Inhibition

The role of IL-6 in the pathogenesis of CAD and ACS is well recognized; therefore, pharmaceutical inhibition could be an effective treatment. Several clinical trials have also associated IL-6 with cardiovascular outcomes. Increased IL-6 levels two days after an ST-elevation MI were associated with adverse cardiac remodeling, microvascular obstruction, and intramyocardial hemorrhage as determined by CMR according to a prospective study that included 170 patients [127]. Canagliflozin Cardiovascular Assessment Study (CANVAS) found that each doubling of IL-6 was associated with a 15% increased risk for cardiovascular and kidney outcomes. Furthermore, every 25% lower level of IL-6 at one year was associated with a 7% lower risk for the same outcomes [128]. Similar findings were also reported from a sub-study of the Stabilization of Atherosclerotic Plaque by Initiation of Darapladib Therapy (STABILITY) trial [129]. These findings have been summarized in a relevant meta-analysis that found that per 1 standard deviation increase in IL-6 levels, there was a significantly increased hazard for nonfatal MI and CAD-related death (HR:1.25 (1.19–1.32)). The same study also showed significant associations of the aforementioned endpoints with IL-18, MMP-9, sCD40L, and TNF-α [130]. Additionally, using participants from the PROspective Multicenter Imaging Study for Evaluation of Chest Pain (PROMISE), IL-6 was associated with MACE, but not with the presence of obstructive CAD. These findings indicate that the role of IL-6 is significant even in patients presenting with MI and non-obstructive coronary arteries (MINOCA) [131]. Patients presenting with MINOCA are characterized by increased markers of inflammation, which could even surpass the ones reported in patients with MI and obstructive coronary arteries [132,133]. These findings may indicate a potential role of IL-6 inhibition even in such patients.

Tocilizumab is a humanized monoclonal antibody that has been shown in preclinical or small clinical studies to have beneficial effects on vascular inflammation and platelet aggregation [134,135,136,137]. In the ASSAIL-MI trial, 199 patients with ST-elevation MI were assigned to either a single dose of tocilizumab or placebo and the primary endpoint was the myocardial salvage index as calculated with MRI. The first group had a higher myocardial salvage index indicating increased myocardial salvage after an MI [74]. In addition, there was no significantly increased risk of infections in the treatment group. However, the study population was low, the follow-up period was not sufficient, and there are concerns about the adverse effects on patients with rheumatologic diseases.

Ziltivekimab is a human monoclonal antibody targeting the IL-6 ligand that was developed specifically for the treatment of cardiovascular diseases. Targeting the ligand rather than the receptor leads to possibly lower clinically effective doses, which should have fewer adverse effects [138]. Ziltivekimab has shown favorable results, especially in patients with chronic kidney disease, which is a common comorbidity of cardiovascular diseases [138,139]. RESCUE was a randomized, double-blind, phase-2, multicenter trial that included patients with moderate to severe chronic kidney disease and hs-CRP above 2 mg/L. There were dose-dependent reductions in hs-CRP, fibrinogen, haptoglobin, secretory phospholipase A2, and lipoprotein (a) levels, indicating a favorable anti-inflammatory and anti-thrombotic profile [75]. A secondary analysis of the RESCUE trial also showed a reduced neutrophil-to-lymphocyte ratio, indicating that ziltivekimab may disrupt several inflammatory pathways involved in atherogenesis [140].

As a consequence of these promising data, a phase-3 randomized controlled trial known as ZEUS is currently underway and will compare ziltivekimab to placebo among more than 5000 patients with evidence of chronic cardiovascular disease, chronic kidney disease, and elevated hs-CRP. This trial will try to determine whether ziltivekimab reduces cardiovascular event rates [141].

### 3.6. Other Anti-Inflammatory Therapies

The effect of TNF-α inhibitors, such as infliximab, etanercept, and adalimumab, in atherosclerosis has been evaluated in preclinical studies or in patients with rheumatological diseases where they have shown anti-inflammatory properties [142]. Infliximab reversed TNF-α induced endothelial dysfunction, but the effect was reversed by the presence of anti-infliximab antibodies. This mechanism was hypothesized to be significant in promoting atherosclerosis in some patients with rheumatologic diseases who were on treatment with infliximab [143]. Etanercept could prevent TNF-induced endothelial cell apoptosis, while Adalimumab could improve endothelial function and inflammation in patients with psoriasis [144,145]. A meta-analysis in patients with rheumatoid arthritis found that TNF-α inhibitors can reduce aortic stiffness [146]. In the same population group, another meta-analysis of epidemiological and randomized control studies found that patients on anti-TNF therapy had reduced risk for cardiovascular events. It is worth noting, however, that this effect was not significant when only randomized control trials were included in the meta-analysis [147]. Data regarding the effect of TNF-α inhibitors on the cardiovascular risk of patients with psoriasis are insufficient, according to a relevant systematic review [148].

Anakinra is a recombinant IL-1 receptor antagonist used as a treatment in several inflammatory diseases and could potentially reduce vascular inflammation in patients with CAD. There are data from murine models that anakinra improves endothelial function [149]. In addition, anakinra improved endothelial function in patients with rheumatoid arthritis and CAD and reduced the leucocyte count in patients with myocardial infarction [150,151]. However, according to data from two small randomized studies, anakinra did not reduce the risk for cardiovascular events [152].

Methotrexate is an anti-inflammatory agent used in various autoimmune and autoinflammatory diseases and is believed to reduce vascular inflammation by inhibiting IL-1, IL-6, and TNF-α [153]. However, low-dose methotrexate failed to reduce the levels of CRP, IL-6, and IL-1b in a double-blind placebo-controlled randomized trial that included more than 4500 patients with established CAD and metabolic syndrome or diabetes. Methotrexate also failed to reduce the risk of the composite of nonfatal myocardial infarction, nonfatal stroke, or cardiovascular death [72]. It is worth noting, however, that in this study, the basal median CRP levels were 1.5 mg/L, indicating a relatively low level of subclinical inflammation in the study population.

Other cytokines have also been utilized in preclinical studies as potential targets for anti-inflammatory treatment. In one such study, repeated gene transfers of a soluble mutant of IFN-γ receptors decreased lipid and macrophage accumulation and increased fibrotic area. It also reduced the expression of pro-inflammatory cytokines, chemokines, adhesion molecules, and matrix metalloproteinases [154]. IL-10 is an anti-inflammatory protein; therefore, its presence in the atheromas could potentially benefit. Initial attempts used viruses to induce gene transfer, but more recently, targeted delivery by nanocarrier succeeded in reducing IL-1β levels and the atherosclerotic plaque size in apoE^−/−^ mice [155,156].

The NLRP3 inflammasome is significant for the production of pro-inflammatory cytokines, and as such, its inhibition is a potential therapeutic target [157]. MCC950 can specifically inhibit the NLRP3 inflammasome it has reduced atherogenesis in an apoE^−/−^ mouse model and has reduced myocardial fibrosis in a mouse model of myocardial infarction [157,158,159].

CCL-2 and CCL-5 (RANTES) are chemokines implicated in the pathophysiology of coronary artery disease. A meta-analysis of 14 preclinical studies testing 11 different CCL-2 or CC receptor 2 (CCR2) inhibitors found that CCL2/CCR2 blockade reduced atherosclerotic lesion size and macrophage accumulation and modified VSMC proliferation. The effects of CCL2/CCR2 inhibition on lesion size correlated with reductions in plaque macrophage accumulation [160]. Inhibition of the CCL-5 receptor in mouse model decreased leukocyte infiltrations and increased collagen content of the atheromas. In addition, it reduced the local expression of VCAM-1, ICAM-1, MCP-1, and TNF-α, among others [161,162].

Recently, ROS-targeting nanoparticles were used in apoE^−/−^ mice and displayed targeted delivery on the vulnerable plaques as determined by magnetic resonance/fluorescence dual-modality imaging. They also inhibited the progress of atherosclerosis, vascular inflammation, endothelial cell apoptosis as well as the formation of foam cells [163]. Nanoparticles have emerged as a diagnostic imaging tool and as a carrier for novel anti-inflammatory therapies. The non-invasive imaging nanoprobes include optical nanoprobes, photoacoustic nanoprobes, magnetic resonance nanoprobes, positron emission tomography nanoprobes, and other dual- and multi-modality imaging nanoprobes and allow efficient recognition of early-stage atherosclerotic plaques [164]. Early recognition is significant for improving long-term cardiovascular outcomes since the inflammatory mechanisms begin early in the process of the disease and could be more effectively targeted.

## 4. Clinical Implications

The anti-inflammatory properties of aspirin and statins are in part responsible for the favorable cardiovascular profile and the clinical application of these therapies, which have been proven to reduce mortality in patients with CAD or those at increased risk for future events. Moreover, according to the 2021 guidelines on cardiovascular disease prevention by the European Society of Cardiology, low-dose colchicine (0.5 mg once daily) may be considered in secondary prevention of cardiovascular disease, particularly if recurrent events occur under optimal therapy [165]. Recently the CANTOS study showed for the first time that isolated treatment targeting inflammation (IL-1β inhibition) can reduce the risk of cardiovascular events and may be useful in patients post-ACS. However, the increased risk for non-cardiac complications and the non-significant effect on all-cause mortality has raised the need for the development of other novel therapeutic strategies as well as for an improved selection of patients more likely to respond to anti-inflammatory therapies. Currently, a large randomized trial examining the therapeutic potential of IL-6 inhibition is underway, and, in addition, other novel therapies have emerged. The findings of such studies can potentially establish the utilization of therapies specifically targeting inflammation for the reduction of cardiovascular risk in patients with established CAD.

## 5. Conclusions

Inflammation plays a significant role in the pathogenesis of atherosclerosis as evidenced by the data of preclinical studies and large epidemiological trials. The pathophysiological association between inflammation and atherosclerosis is very complex and many mediators are included. The most important mediators of this association are the cytokines and, specifically, IL-1β and IL-6 chemokines, IFN-γ, and TNF-α. Large randomized clinical studies and meta-analyses have shown that anti-inflammatory therapies have a favorable efficacy profile and can reduce the hazard of cardiovascular events. However, the results of randomized trials of therapies specifically targeting inflammation are controversial, and, therefore, further studies are needed to elucidate this association in the future.

## Figures and Tables

**Table 1:** Main mechanism of action of the most common anti-inflammatory therapies.

Anti-Inflammatory Therapy	Action
Aspirin	COX-inhibition Inhibition of NF-κΒ pathway Activation of Thromboxane prostanoid receptor IL-6 and TNF-α reduction Macrophage-colony-stimulating factor inhibition Increased activity of NO synthase Increased the formation of 15-epi-LXA4 Decreased MRP8/14 levels
Statins	Reduction of endothelin-1 Reduction of asymmetrical dimethyl arginine Reduction of NO synthesis Reduction of TNF-α, IL-6, IFN-γ and MCP-1 levels Increase in IL-10 levels Increased macrocalcification
Colchicine	Inhibition of tubulin polymerization Reduction of chemotaxis Decreased expression of adhesion molecules Inhibition of NF-κΒ pathway Reduced formation of the inflammasome
IL-1β inhibitors	Inhibition of NF-κΒ pathway Reduction of IL-6 and TNF-α Reduced expression of ICAM-1, VCAM-1, P-selectin, E-selectin, MCP-1 Reduced transmigration of inflammatory cell
IL-6 inhibitors	Reduction of vascular inflammation Reduced expression of adhesion molecules Reduced production of TNF-α, IL-18, and IFN-γ Reduced production of fibrinogen Reduction of MMP-9
TNF-α inhibitors	Reduced endothelial cell apoptosis Reduced expression of ICAM-1, VCAM-1 and MCP-1
IL-1 receptor antagonist	Reduction of vascular NADPH oxidase Reduced activation of NF-Κb Reduced NO synthesis
gene transfers of mutant IFN-γ receptors	Decreased macrophage accumulation Reduced VSMC proliferation Reduced expression of cytokines, adhesion molecules, and MMPs
NLRP3 inflammasome inhibitors	Reduced expression of adhesion molecules Reduced macrophage accumulation
CCLR/CCR2 inhibitors	Reduced macrophage accumulation Reduced VSMC proliferation Improved endothelial function
CCL5 inhibitors	Reduced local expression of VCAM-1, ICAM-1, MCP-1 Reduced leukocyte infiltrations of the atheromas Reduced TNF-α
ROS-targeting nanoparticles	Reduced endothelial cell apoptosis Reduced vascular inflammation Reduced formation of foam cells

COX: cyclooxygenase; ICAM-1: intercellular adhesion molecule; IFN: interferon; IL: interleukin; MCP-1: monocyte chemoattractant protein-1; MMP: matrix metalloproteinase; MRP: myeloid related protein; NADPH: nicotinamide adenine dinucleotide phosphate NO: nitric oxide; NF-Κb: nuclear factor kappa-light-chain-enhancer of activated B cells TNF-α: tumor necrosis factor-α; VCAM-1: vascular cell adhesion molecule; VSMC: vascular smooth muscle cell.

**Table 2:** Most significant studies of anti-inflammatory therapies.

First Author/ Study Name	Anti-Inflammatory Therapy	Type of Study	Date	Main Findings
Ridker et al. [64]	Aspirin	Case control study	1997	Aspirin reduced the risk of MI in patients in the highest quartile of hs-CRP but not in those in the lowest quartile
Kronish et al. [65]	Aspirin	Prospective cohort	2010	Adherence to aspirin inversely correlated with hs-CRP levels
Ridker et al. JUPITER trial [66]	Statins	Randomized trial	2008	Rosuvastatin reduced CRP levels by 37% Rosuvastatin reduced the risk for cardiovascular events (HR: 0.56; 95% CI, 0.46 to 0.69; *p* < 0.001) and all-cause mortality
Sahebkar et al. [67]	Statins	Meta-analysis	2015	Statin therapy reduces endothelin-1 levels
Arabi et al. [68,69]	Statins	Meta-analysis	2022	Statin and ezetimibe combination therapy reduce levels of hs-CRP and various cytokines
Tardif et al. COLCOT trial [70]	Colchicine	Randomized trial	2019	Colchicine reduced the risk for cardiovascular mortality in patients recruited within 30 days after a MI (HR, 0.77; 95% CI, 0.61 to 0.96; *p* = 0.02)
Nidorf et al. LoDo-Co-2 trial [71]	Colchicine	Randomized trial	2020	Colchicine reduced the risk for cardiovascular events in patients with chronic CAD (HR: 0.69; 95% CI 0.57 to 0.83; *p* < 0.001)
Ridker et al. CIRT trial [72]	Methotrexate	Randomized trial	2019	Methotrexate did not reduce the risk for the primary endpoint (HR: 0.96; 95% CI 0.79 to 1.16; *p* = 0.67)
Ridker et al. CANTOS trial [73]	Canakinumab	Randomized trial	2017	Canakinumab achieved a dose-dependent reduction of CRP and a reduction of the hazard for nonfatal MI/stroke or cardiovascular death in patients with previous MI and hs-CRP > 2 mg/L (HR: 0.85 (95% CI, 0.74 to 0.98; *p* = 0.021 in the 150 mg dose group) Canakinumab associated with increased risk for fatal infection
Broch et al. ASSAIL-MI [74]	Tocilizumab	Randomized trial	2021	Tocilizumab increased myocardial salvage in patients with ST-elevation MI
Ridker et al. RESCUE trial [75]	Ziltivekimab	Randomized trial	2021	Ziltivekimab achieved dose-dependent reduction (77–92%) of hs-CRP levels in patients with increased cardiovascular risk, chronic kidney disease, and hs-CRP > 2 mg/L

CAD: coronary artery disease; CI: confidence interval; CRP: C-reactive protein; HR: hazard ratio; MI: myocardial infarction.

## Data Availability

Not applicable.
